# MAE4, an eLtaS monoclonal antibody, blocks *Staphylococcus aureus* virulence

**DOI:** 10.1038/srep17215

**Published:** 2015-11-24

**Authors:** Yu Liu, Jiannan Feng, Qiang Lu, Xin Zhang, Yaping Gao, Jun Yan, Chunhua Mu, Yan Hei, Ming Lv, Gencheng Han, Guojiang Chen, Peng Jin, Weiguo Hu, Beifen Shen, Guang Yang

**Affiliations:** 1 Beijing Institute of Basic Medical Sciences, Beijing, China; 2 State key Laboratory of Toxicology and Medical Countermeasures, Beijing Institute of Pharmacology and Toxicology, Beijing 100850, China; 3 Institutes of Biomedical Sciences, Fudan University, Shanghai, China; 4 People’s Armed Police Corps General Hospital, Beijing, China

## Abstract

*Staphylococcus aureus* causes a wide range of infectious diseases. Treatment of these infections has become increasingly difficult due to the widespread emergence of antibiotic-resistant strains; therefore, it is essential to explore effective alternatives to antibiotics. A secreted protein of *S. aureus*, known as eLtaS, is an extracellular protein released from the bacterial membrane protein, LtaS. However, the role of eLtaS in *S. aureus* pathogenesis remains largely unknown. Here we show eLtaS dramatically aggravates *S. aureus* infection by binding to C3b and then inhibiting the phagocytosis of C3b-deposited *S. aureus*. Furthermore, we developed a monoclonal antibody against eLtaS, MAE4, which neutralizes the activity of eLtaS and blocks staphylococcal evasion of phagocytosis. Consequently, MAE4 is capable of protecting mice from lethal *S. aureus* infection. Our findings reveal that targeting of eLtaS by MAE4 is a potential therapeutic strategy for the treatment of infectious diseases caused by *S. aureus.*

*Staphylococcus aureus* is a major human pathogen that causes a wide range of illnesses, ranging from mild skin infections to bacteremia, sepsis, and endocarditis^1^,^2^,^3^,^4^,^5^. Treatment of *S. aureus* infections has become increasingly difficult because of the prevalence of antibiotic-resistant strains such as methicillin-resistant *S. aureus* (MRSA) strains^6^,^7^,^8^. To combat the rapid emergence of antibiotic-resistant strains, several alternative therapeutic options are being explored, including the development of inhibitors of virulence factors, such as α-hemolysin, Protein A, and AgrA^9^,^10^,^11^. AR-301, the human monoclonal antibody directed against secreted α-hemolysin, has been approved for a clinical trial in Europe to treat intensive care unit patients with severe *S. aureus* pneumonia infections^12^.

A membrane-embedded enzyme known as LtaS, which contains five N-terminal transmembrane helices followed by a large extracellular domain (eLtaS), is required for *S. aureus* growth and synthesis of lipoteichoic acid (LTA)^13^,^14^,^15^,^16^. Richter *et al.* previously identified a small molecule inhibitor of LtaS that reduced the severity of infections by inhibiting *S. aureus* growth^16^. It has been established that LtaS protein is processed during bacterial growth and that the extracellular domain is released following hydrolysis of residues Ala^215^-Leu^216^-Ala^217^ by the peptidase SpsB^15^. However, no LTA synthase activity has been identified within the eLtaS domain and its function is still unclear^15^.

In the present study, we demonstrated that eLtaS mediates phagocytic evasion of *S. aureus* via binding to the complement component C3b. Furthermore, we have developed a neutralizing monoclonal antibody against eLtaS that blocks eLtaS-mediated evasion of phagocytosis and consequently protects mice from *S. aureus* infection.

## Results

### eLtaS aggravates *S. aureus* infection

**Figure 1:**
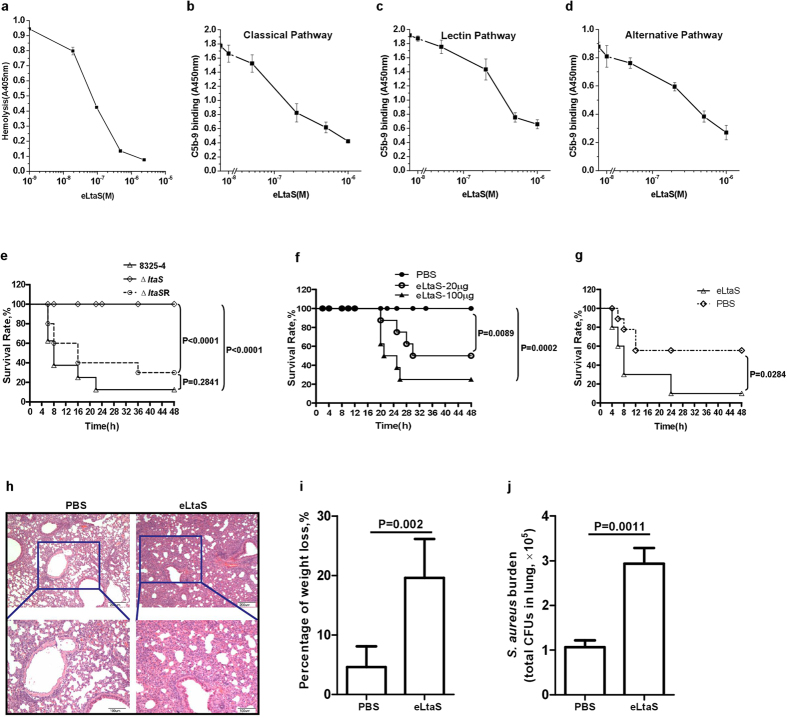
eLtaS aggravates *S. aureus* infection. (**a**) eLtaS inhibits the hemolysis of red blood cells (RBCs) in a concentration-dependent manner. Opsonized sheep erythrocytes (2 × 10^7^) were incubated with 25% pre-cleared normal human serum in the presence of eLtaS at the concentrations indicated for 30 min at 37 °C. The samples were centrifuged, and the absorbance of the supernatants was measured at 405 nm. (**b–d**) eLtaS inhibits C5b-9 formation via the classic (2% serum; (**b**)), lectin (10% serum; (**c**)), and alternative (20% serum; (**d**)) pathways in a concentration-dependent manner. Serum samples were pre-incubated with eLtaS at the concentrations indicated. The serum and eLtaS mixture was added to plates coated with fibrinogen immune complex (classic pathway), immobilized mannan (lectin pathway), or LPS (alternative pathway). The formation of C5b-9 was detected using an anti-C5b-9 antibody. Data are presented as the mean ± SD. **e-g.** Survival rate of mice challenged with different *S. aureus* strains in the acute peritoneal infection model. *S. aureus* 8325-4 (5 × 10^8^ cfu/mouse), Δ*ltaS* (2 × 10^9^ cfu/mouse) or Δ*ltaS*R (5 × 10^8^ cfu/mouse) was injected into the peritoneal cavity of CD-1 mice (**e**). CD-1 mice were intraperitoneally injected with Δ*ltaS* (2 × 10^9^ cfu/mouse) in the presence of eLtaS (**f**). *S. aureus* 8325-4 (2 × 10^8^ cfu/mouse) was injected in the presence of eLtaS (**g**). The survival rate was calculated at different time points post challenge. Data are presented as percentages of surviving mice (n = 8). Survival curves were determined using the Kaplan-Meier method and compared using the log-rank test. (**h–j**) Determination of the role of eLtaS in a sublethal murine pneumonia infection model. CD-1 mice were infected with *S. aureus* 8325-4 cells (1 × 10^7^ cfu/mouse) intratracheally in the presence of eLtaS protein. Lung sections were obtained at 72 h post challenge and stained with hematoxylin-eosin (**h**). Weight loss was monitored for 72 h post infection (**i**). Lung bacterial burdens were determined at 24 h after challenge (**j**).

Previously, we reported that the supernatant of a *S. aureus* RNase III mutant strain (Δrnc) contained reduced levels of most proteins^17^. However, the extracellular proteins of Δrnc were more effective at blocking complement-mediated red blood cell lysis than those of its parent strain, *S. aureus* 8325-4 (Fig. S1a,b). To identify the proteins involved in blocking sheep red blood cell lysis mediated by complement system, we compared the extracellular protein profiles of Δrnc with those of *S. aureus* 8325-4. As shown in Fig. S2a,b, two proteins, LytM and eLtaS, were present at higher levels in the supernatants of Δrnc strain than in the supernatants of the *S. aureus* 8325-4 strain, as determined by mass spectrometry. Similar results were obtained by western blotting (Fig. S2c). We then examined the effect of both LytM and eLtaS on complement-mediated red blood cell lysis and found that eLtaS, but not LytM, was responsible for this effect (Fig. 1a and S2d).

The complement system is a family of proteins and proteolytic fragments with multiple roles in both innate and acquired immunity, including direct killing of foreign cells and regulation of other effectors of the immune response^18^,^19^. The complement system can be activated by three separate pathways: the classical pathway (CP), alternative pathway (AP), and lectin pathway (LP)^20^. Formation of the membrane attack complex (MAC; C5b-9) is common to all three complement pathways^21^. We examined the effect of eLtaS on the formation of C5b-9 according to the methods described by Jongerius *et al.*^22^ and demonstrated that eLtaS inhibited C5b-9 formation in all three pathways (Fig. 1b-d).

Given the importance of the complement system in host protection from pathogens, we then considered whether eLtaS could aggravate *S. aureus* infection. The gene encoding LtaS was deleted from the *S. aureus* 8325-4 genome to generate an *ltaS*-null strain (Δ*ltaS*). The effect of the mutant strain was evaluated in a murine model of acute peritoneal infection. *S. aureus* 8325-4 or Δ*ltaS* cells were injected into the peritoneal cavity of CD-1 mice, and the survival of the mice was recorded over 48 h. Injection of wild-type *S. aureus* 8325-4 and the *ltaS*-complemented strain (Δ*ltaS*R) (5 × 10^8^ cfu/mouse) resulted in a significant increase in mortality at 48 h. However, injection of Δ*ltaS* (2 × 10^9^ cfu/mouse) was non-lethal to CD-1 mice at the same time point (Fig. 1e).

The impaired pathogenicity of (ltaSltas may be attributed to retarded S. aureus growth as a result of LtaS deficiency, and not specifically to the deficiency of the extracellular domain^14^. Hence, we injected Δ*ltaS* (2 × 10^9^ cfu/mouse) into the peritoneal cavity of CD-1 mice in the presence of various amounts of eLtaS protein (20–100 μg/mouse). We found that co-injection of eLtaS significantly decreased the survival rate of the mice (Fig. 1f, P = 0.0089), but injection of eLtaS (100 μg/mouse) alone was harmless (Fig. S3). Furthermore, we demonstrated that injection of *S. aureus* 8325-4 (2 × 10^8^ cfu/mouse) together with eLtaS significantly decreased the survival rate (Fig. 1g, P = 0.0284). The pathogenic role of eLtaS was also determined in a sub-lethal murine pneumonia infection model. Histopathological examination of the mouse lungs showed that eLtaS increased capillary congestion and thickness of the alveolar wall (Fig. 1h). Meanwhile, eLtaS also led to more weight loss in mice at 72 h (Fig. 1i) and increases lung bacterial burden at 24 h (Fig. 1j). These results suggest that *S. aureus*–secreted eLtaS is an important virulence factor that dramatically aggravates infection.

### eLtaS binds to C3b

**Figure 2:**
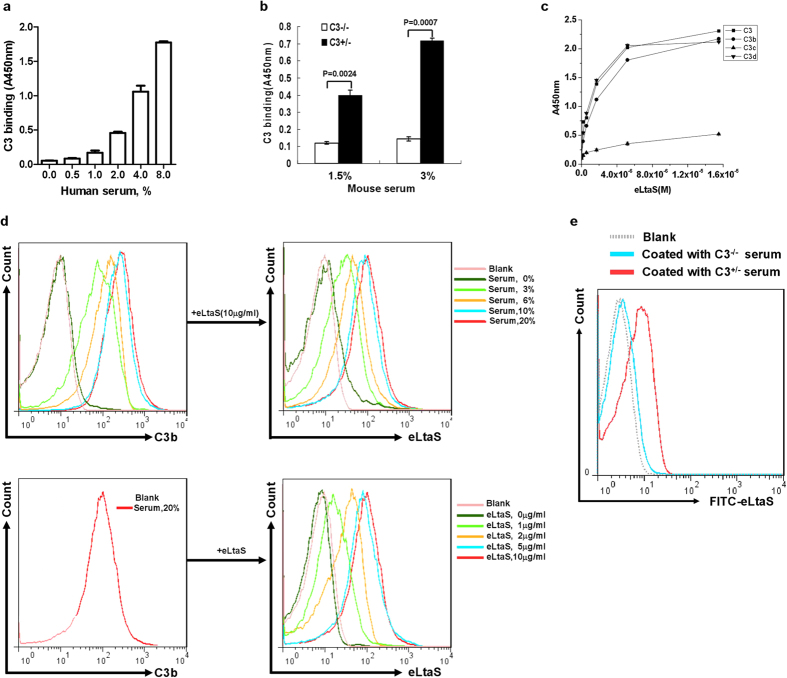
eLtaS binds to C3b. (**a**) eLtaS binds to C3 from human serum in a concentration-dependent manner. eLtaS was coated onto a 96-well plate and human serum was added at various concentrations. Bound C3 was detected using an anti-C3 antibody. Data are representative of three independent experiments and shown as the mean ± SD. (**b**) eLtaS binds to murine C3. Serum (3% and 1.5%) from C3^ + /−^ or C3^−/−^ mice was added to an eLtaS coated 96-well plate. C3 was detected using an anti-C3 antibody. Data are representative of three independent experiments and shown as the mean ± SD. Significant differences between groups were evaluated using a two-tailed Student’s t test. (**c**) eLtaS binds to C3, C3b, and C3d, but not C3c. Human complement C3 and its fragments, C3b, C3c, and C3d, were coated (1 μg/well) onto a 96-well plate. Bound eLtaS was detected using an anti-eLtaS antibody. Data are presented as the mean ± SD of three independent experiments. (**d**) eLtaS binds to C3b-deposited *S. aureus. S. aureus* 8325-4 was incubated with the indicated concentrations of human serum for 15 min. After washing with PBS, *S. aureus* was incubated with eLtaS for a further 30 min. Deposited C3b and bound eLtaS were detected by FCM with anti-C3b and anti-His antibodies. (**e**) eLtaS binds to C3b. Serum was isolated from C3^+/−^ and C3^−/−^ C57BL/6 mice and incubated with *S. aureus* 8325-4 FITC-labeled eLtaS protein was added and specific binding was detected by FCM.

Given that eLtaS inhibits MAC formation and that C3 is the central component protein in all three complement pathways, we investigated the interaction between eLtaS and C3. Binding of eLtaS to C3 from both human and mouse serum was detected by capture ELISA (Fig. 2a,b), indicating a species-independent interaction. This interaction was not observed with serum from C3-deficient (C3^−/−^) C57BL/6 mice (Fig. 2b).

Two forms of C3 convertase (C4b2a and C3bBb) cleave C3 into C3a and C3b. C3b is further cleaved into C3c and C3d by Factor I^20^,^23^,^24^. To identify the fragment responsible for eLtaS binding, a capture ELISA was performed with C3 and its proteolytic fragments, C3b, C3c, and C3d, as coating antigens. eLtaS was found to bind the C3d domain of C3b, but not C3c (Fig. 2c). The relative affinity of eLtaS for human C3 and C3b was determined respectively (Table S1).

C3b binds covalently to bacterial surfaces through a reactive intramolecular thioester in the C3d domain of the α-chain, either to hydroxyl groups via an ester linkage or to primary amino groups via an amide bond^25^,^26^. Three strains of *S. aureus* (8325-4, Newman, and 04018) were incubated with increasing concentrations of human serum for 15 min and the deposition of C3b was detected by flow cytometry (FCM) using an anti-C3b-FITC antibody. The percentage of C3b-deposited *S. aureus* increased in a serum concentration-dependent manner (Fig. 2d and S4). Using an eLtaS-specific antibody, we further demonstrated that, following incubation, eLtaS bound to C3b deposited on the bacterial surface (Fig. 2d). Sera from C3^+/−^ and C3^−/−^ C57BL/6 mice were incubated with *S. aureus* 8325-4 prior to addition of FITC-labeled eLtaS. Analysis by FCM showed that eLtaS only bound to *S. aureus* incubated with serum from C3^+/−^ C57BL/6 mice and not from C3^−/−^ C57BL/6 mice (Fig. 2e), confirming that eLtaS is able to bind C3b deposited on the bacterial surface.

### eLtaS attenuates phagocytosis of C3b-deposited *S. aureus*

Immune effector cells, such as neutrophils, target C3b-deposited *S. aureus* for destruction^27^. We considered whether eLtaS blocked the process of C3b deposition in the three complement activation pathways. *S. aureus* Efb (extracellular fibrinogen binding protein), which is known to inhibit C3b deposition in the AP, was used as a positive control^28^,^29^. As expected, Efb inhibited C3b deposition in AP. However, deposition of C3b was not blocked by eLtaS in any of the pathways (Fig. S5a–c), suggesting that the interaction site of eLtaS on C3d was different from that of Efb. We further demonstrated that eLtaS could not inhibit binding between C3d and Efb. Similarly, the interaction between C3d and eLtaS was not interrupted by Efb (Fig. S6a,b). The ability of eLtaS to block C3b deposition was tested by incubating human serum with *S. aureus* 8325-4 in the presence or absence of eLtaS. As shown in Fig. S7, the level of C3b deposited on the *S. aureus* surface was unaffected by the presence of eLtaS.

**Figure 3:**
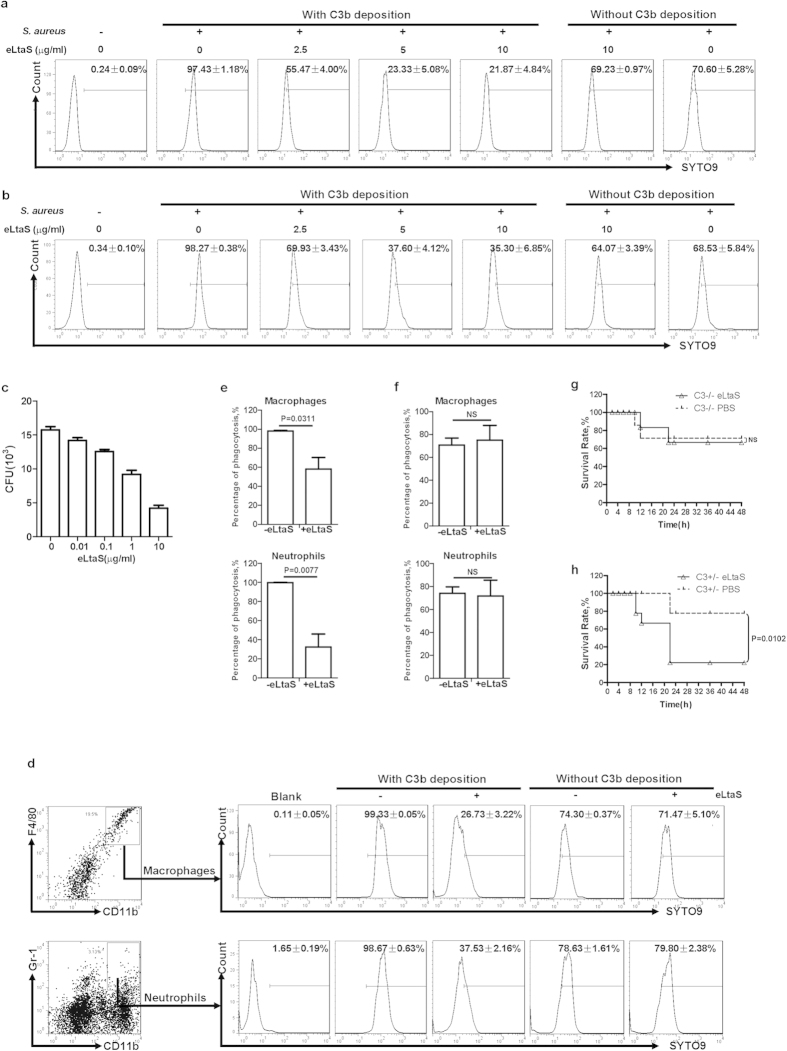
eLtaS attenuates C3b-mediated engulfment of *S. aureus* by phagocytes. (**a,b**) Determination of the engulfment of *S. aureus* by neutrophils through FCM. SYTO9-labeled *S. aureus* was incubated with human serum (**a**) or murine serum (**b**). The complexes were then incubated in the presence of eLtaS prior to addition of human (**a**) or murine (**b**) leukocytes. The percentage of human or murine neutrophils that were SYTO9-positive, indicating engulfment of *S. aureus,* was determined by FCM. (**c**) Determination of the engulfment of *S. aureus* by human neutrophils mediated by C3b deposition on the bacterial surface. C3b-deposited *S. aureus* 8325-4 was incubated with different concentrations of eLtaS prior to incubation with human neutrophils. The engulfed *S. aureus* 8325-4 cells were counted on a THA plate. Data are representative of three independent experiments and shown as the mean ± SD. (**d**) Determination of the engulfment of *S. aureus* by murine peritoneal macrophages and neutrophils *in vitro*. Murine peritoneal cavity cells were isolated and incubated with SYTO9-labeled *S. aureus* in the presence of eLtaS. Phagocytosis was determined by FCM using anti-CD11b-APC (Mac-1), anti-F4/80-PE, and Gr-1-PerCP (Ly-6 G). (**e,f**) Evaluation of the engulfment of *S. aureus* by murine peritoneal phagocytes *in vivo* in C3^+/−^ C57BL/6 (**e**) and C3^−/−^ C57BL/6 (**f**) mice. Peritoneal cavity cells were isolated 30 min after intraperitoneal injection of SYTO9-labeled *S. aureus* 8325-4 (5 × 10^8^ cfu/mouse) in the presence of eLtaS. The percentage of neutrophils and macrophages that had engulfed *S. aureus* was determined by FCM. Data are representative of three independent experiments and shown as the mean ± SD. Significant differences between groups were evaluated using a two-tailed Student’s t test. (**g,h**) The role of eLtaS in the mortality of C3-deficient mice. C3^−/−^ (**g**) and C3^+/−^ C57BL/6 (**h**) mice were injected intraperitoneally with *S. aureus* 8325-4 cells (5 × 10^8^ cfu/mouse for C3^+/−^ C57BL/6 mice, 1 × 10^8^ cfu/mouse for C3^−/−^ C57BL/6 mice) in the presence of eLtaS. The survival rate was calculated at different time points post challenge. Data are presented as the percentage of mice surviving. Survival curves were determined using the Kaplan-Meier method and compared using the log-rank test (n = 8) (NS, non-significant).

To determine whether eLtaS attenuates phagocytosis of C3b-deposited *S. aureus* by neutrophils, we incubated SYTO9-labeled *S. aureus* 8325-4 with fresh human or murine serum and then added human or murine leukocytes. As neutrophils constitute more than 90% of granulocytes, we detected the proportion of SYTO9-positive granulocytes that had engulfed *S. aureus* by FCM. We found an increased percentage of SYTO9-positive granulocytes in the presence of serum, and this increase was reduced in a concentration-dependent manner in the presence of eLtaS (Fig. 3a,b). Furthermore, human neutrophils were isolated from peripheral blood^30^ and incubated with C3b-deposited *S. aureus* 8325-4 in the presence of eLtaS. The number of *S. aureus* cells engulfed by neutrophils was found to decrease with increasing amounts of eLtaS (Fig. 3c).

Murine peritoneal cavity cells were isolated and incubated with SYTO9-labeled *S. aureus*, and the percentage of phagocytes, including neutrophils and macrophages, that engulfed *S. aureus* was determined by FCM. We found that eLtaS attenuated the engulfment of C3b-deposited *S. aureus* by peritoneal phagocytes (Fig. 3d).

We then studied the effect of eLtaS on phagocytosis of *S. aureus in vivo* using C3^+/−^ C57BL/6 mice. Mouse peritoneal cavity cells were isolated after intraperitoneal injection of SYTO9-labeled *S. aureus* in the presence or absence of eLtaS. We found that the percentage of phagocytes that had engulfed *S. aureus* was decreased in the presence of eLtaS (Fig. 3e). However, eLtaS had no significant inhibitory effect on the phagocytosis of *S. aureus* by peritoneal phagocytes of C3^−/−^ C57BL/6 mice (Fig. 3f). These results further demonstrated that eLtaS attenuated the engulfment of *S. aureus* by phagocytes through interaction with C3.

We then determined the pathogenic effect of eLtaS in an acute peritoneal infection murine model. eLtaS did not aggravate *S. aureus* infection in C3^−/−^ C57BL/6 mice (Fig. 3g), but injection of eLtaS in heterozygous C3^+/−^ C57BL/6 mice resulted in a decreased survival rate (Fig. 3h).

### Monoclonal antibody 4 against eLtaS (MAE4) prevents *S. aureus* infection

**Figure 4:**
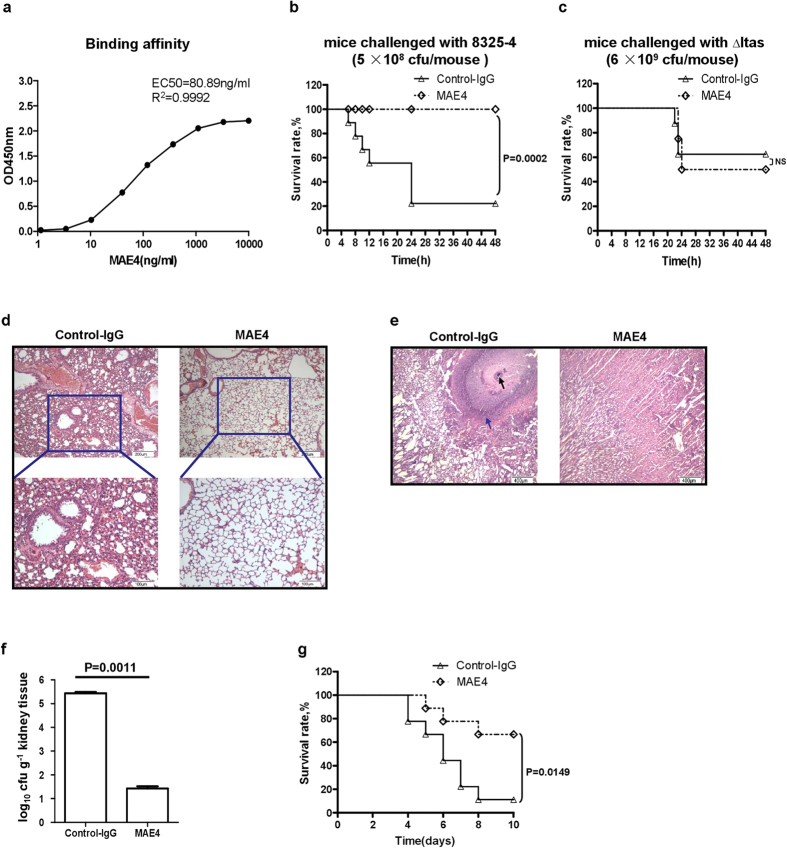
The eLtaS-specific monoclonal antibody MAE4 efficiently prevents *S. aureus* infection. (**a**) The binding affinity of MAE4 to eLtaS was determined by ELISA. eLtaS protein was coated onto 96-well plates in the presence of a serial dilution of MAE4. Bound IgG was detected using peroxidase (HRP)-conjugated goat anti-mouse IgG. (**b,c**) Assessment of the anti-infective effect of MAE4 in a murine model of acute peritoneal infection. MAE4 (100 μg/mouse) was injected into the peritoneal cavity 2 h prior to challenge with *S. aureus* 8325-4 (5 × 10^8^ cfu/mouse) (**b**) or Δ*ltaS* (6 × 10^9^ cfu/mouse) (**c**). The survival rate was measured at different time points post challenge. Data are presented as the percentage of mice surviving. Survival curves were determined using the Kaplan-Meier method and compared using the log-rank test (n = 8). (**d**) MAE4 protects mice from *S. aureus* infection in a pneumonia infection model. CD-1 mice were injected intratracheally with *S. aureus* 8325-4 cells (1 × 10^7^ cfu/mouse). MAE4 IgG (100 μg/mouse) was injected intramuscularly into the left hind leg at 30 min, 24 h, and 48 h post infection. Lung sections were taken 72 h post challenge and stained with hematoxylin-eosin. (**e–g**) Determination of the effect of MAE4 in a sublethal murine model of intravenous challenge. MAE4 IgG (100 μg/mouse) was injected into the peritoneal cavity of BALB/c mice 2 h prior to intravenous challenge with *S. aureus* Newman (1 × 10^7^ cfu/mouse). Kidneys were examined by histopathology for internal abscesses 5 days post infection. Staphylococcal abscess (black arrow) with a central concentration of staphylococci (blue arrow) is indicated (**e**). Bacterial colonies were counted at 5 days post infection (**f**). Data are representative of three independent experiments and shown as the mean ± SD. Significant differences between groups were evaluated using a two-tailed Student’s t test. For the lethal challenge model, *S. aureus* Newman cells (1 × 10^8^ cfu/mouse) were administered and survival rates were monitored for 10 days (**g**). Survival curves were determined using the Kaplan-Meier method and compared using the log-rank test (n = 8) (NS, non-significant).

Because eLtaS was found to aggravate *S. aureus* infection, we considered whether an antibody directed against eLtaS could be protective. Mouse monoclonal antibodies to eLtaS were generated according to standard protocols. Seven monoclonal antibodies (MAE1-7) were obtained (Fig. S8a) and one of these, MAE4, was found to block the interaction between eLtaS and C3b (Fig. S8b). MAE4 had an EC_50_ of 80.89 ng/ml (Fig. 4a) and was of an IgG2a isotype (Fig. S8c).

We assessed the protective effect of MAE4 in the murine acute peritoneal infection model. It was found that MAE4 was able to completely rescue mice from lethal *S. aureus* 8325-4 infection (Fig. 4b). As expected, MAE4 had no effect when used in combination with a lethal dose of *S. aureus* Δ*ltaS* (Fig. 4c). These results suggest that MAE4 protected mice from *S. aureus* infection by directly targeting eLtaS. This effect was further studied in a murine model of staphylococcal pneumonia. MAE4 was injected intramuscularly at 30 min, 24 h and 48 h post challenge with *S. aureus* 8325-4. Histopathological examination of the lungs of the mice showed that MAE4 decreased *S. aureus*-induced tissue damage (Fig. 4d). We also assessed the effect of MAE4 in a murine pneumonia infection model with two clinical *S. aureus* strains (*S. aureus* Newman and 04018)^31^,^32^. MAE4 also inhibited infection caused by these two *S. aureus* strains (Fig. S9).

In addition, the protective effect of MAE4 was assessed using a mouse intravenous challenge model. MAE4 was injected (100 μg/mouse) into the peritoneal cavity of BALB/c mice 2 h before intravenous challenge with a sublethal dose of *S. aureus* Newman (1 × 10^7^ cfu/mouse). Five days post infection, the kidneys were examined by histopathology for internal abscesses (Fig. 4e) and the number of bacterial colonies was determined (Fig. 4f). MAE4 was also tested in a lethal challenge model with *S. aureus* Newman (1 × 10^8^ cfu/mouse). Survival rates were monitored for ten days (Fig. 4g). These results further confirm that MAE4 protects mice from *S. aureus* infection.

### MAE4 blocks evasion of phagocytosis mediated by eLtaS

**Figure 5:**
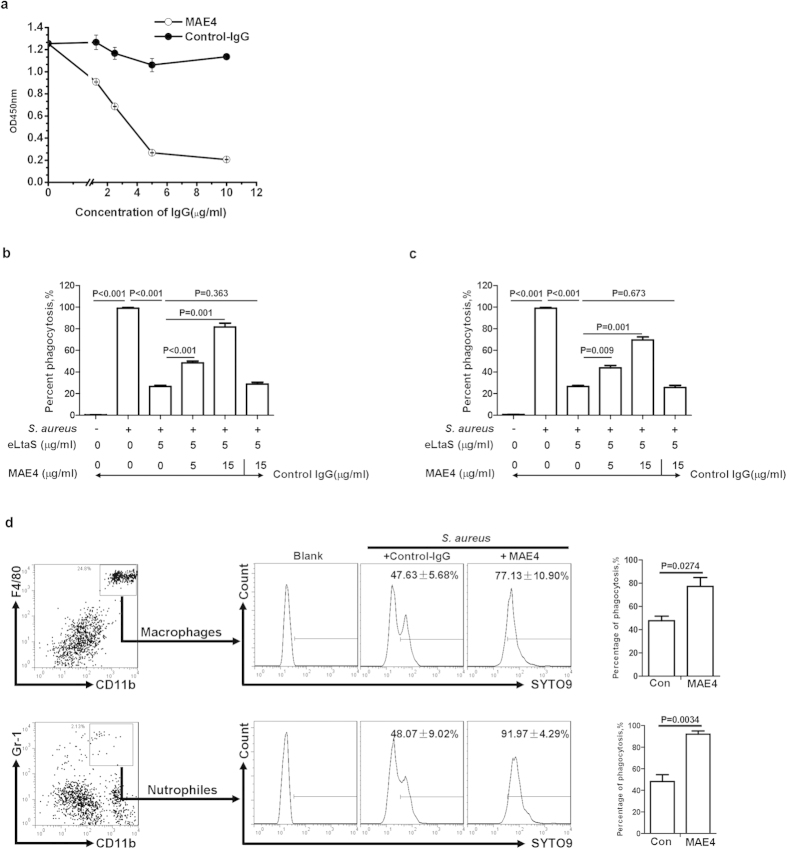
MAE4 blocks eLtaS-mediated evasion of phagocytosis (a). Determination of the effect of MAE4 on the interaction between eLtaS and C3b. C3b was added to an eLtaS-coated 96-well plate in the presence of different concentrations of MAE4. Bound C3b was determined using an anti-C3b antibody. (**b,c**) Analysis of phagocytosis of *S. aureus* by neutrophils *in vitro*. SYTO9-labeled *S. aureus* 8325-4 was incubated with human serum (**b**) or murine serum (**c**). C3b-deposited *S. aureus* was further incubated in the presence of eLtaS with or without MAE4 prior to the addition of human (**b**) or murine (**c**) leukocytes. The percentage of neutrophils that were SYTO9-positive, indicating engulfment of *S. aureus,* was determined by FCM. (**d**) Analysis of the phagocytosis of *S. aureus* by macrophages and neutrophils *in vivo.* MAE4 was injected into the peritoneal cavity 2 h prior to challenge with SYTO9-labeled *S. aureus* 8325-4 (5 × 10^8^ cfu/mouse). Peritoneal cavity cells were isolated, and the percentage of SYTO9-labeled neutrophils and macrophages, indicating engulfment of *S. aureus* was determined by FCM. Data are representative of three independent experiments and shown as the mean ± SD. Significant differences between groups were evaluated using a two-tailed Student’s t test.

We further determined whether MAE4 could neutralize the effect of eLtaS. Using a competitive ELISA, we demonstrated that MAE4 blocked the interaction between eLtaS and C3b in a concentration-dependent manner (Fig. 5a). The effect of MAE4 on eLtaS-mediated evasion of phagocytosis was detected *in vitro* by FCM. We found that eLtaS did not attenuate engulfment of *S. aureus* by human or murine neutrophils in the presence of MAE4 (Fig. 5b,c). We further tested the neutralization effect of MAE4 *in vivo* and demonstrated that treatment with MAE4 increased the percentage of neutrophils and macrophages that engulfed *S. aureus* in the peritoneal cavity (Fig. 5d).

**Figure 6:**
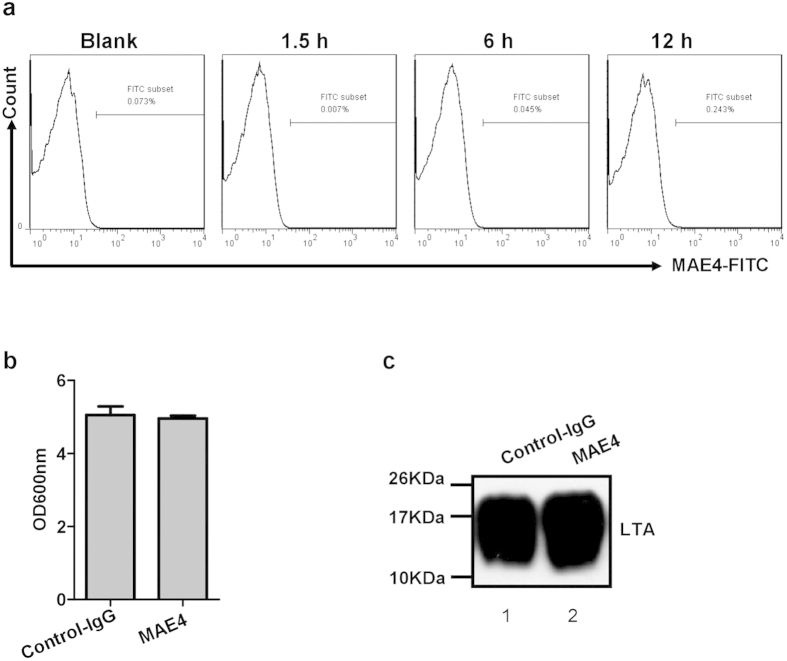
Administration of MAE4 exerts less evolutionary pressure on *S. aureus.* (**a**) MAE4 did not bind to the surface of *S. aureus. S. aureus* 8325-4 cells were cultured and collected at different time points (1.5, 6, or 12 h). MAE4 antibody was incubated with *S. aureus* 8325-4 and binding of MAE4 was detected by FCM. (**b**) MAE4 had no perceptible effect on the growth of *S. aureus. S. aureus* (OD_600_ = 0.1) was cultured with MAE4 or isotype control IgG (50 μg/ml) for 6 h, and the bacterial concentration was determined by measuring the OD_600_. (**c**) MAE4 had no perceptible effect on the production of LTA. Cell-associated LTA from *S. aureus* 8325-4 (2.5 × 10^9^ cfu) cultured with MAE4 or isotype control IgG (50 μg/ml) was extracted, and the level of LTA was analyzed by western blotting using a monoclonal LTA-specific antibody.

Given that LtaS is located on the cell membrane, we tested whether MAE4 could directly bind bacterial cells and affect the growth of *S. aureus in vitro*. Analysis by FCM showed that MAE4 did not bind *S. aureus* cells (Fig. 6a). We further demonstrated that synthesis of LTA and growth of *S. aureus* were unaffected by MAE4 (10 μg/ml) (Fig. 6b,c). The metal-binding domain and substrate-binding domain of LtaS are considered important for LTA synthesis, and mutation of two amino acids (T300 and H347) in these domains decreases the production of LTA^15^. These two amino acids were substituted with alanine to generate two mutant proteins (T300A and H347A). As shown in Fig. S10a,b, MAE4 binding to these two mutant proteins was maintained, indicating that MAE4 only targeted secreted eLtaS protein and had no effect on the activity of membrane-bound LtaS.

## Discussion

The spread of multi-drug-resistant *S. aureus* is an increasingly serious threat to global public health. Global efforts to develop new antibiotics have not been fast enough to combat the evolution of antimicrobial resistance^33–35^. Targeting of virulence factors is regarded as a promising strategy that would apply less selective pressure for the development of bacterial resistance than traditional strategies^36^.

*In vivo*, *S. aureus* bacteria are mainly cleared by phagocytes (neutrophils and macrophages). Phagocytosis is strongly enhanced by the opsonization of bacteria with antibodies and the deposition of complement activation products on the surface of the bacteria^37^,^38^.

In this study, we developed a monoclonal antibody against eLtaS, MAE4, which is capable of blocking eLtaS-mediated evasion of phagocytosis and dramatically reduces *S. aureus* infection. eLtaS, the C-terminal extracellular domain of LtaS, is released from the cell membrane by peptidase SpsB as a soluble peptide whose function was unknown until now^15^. Here, we demonstrate that eLtaS aggravates *S. aureus* infection through binding to C3b.

Complement activation leads to rapid deposition of C3b on the surface of bacteria, promoting phagocytosis of the opsonized bacteria^27^. We found that eLtaS had no effect on the deposition of C3b, but the interaction between eLtaS and C3b resulted in attenuated engulfment of C3b-deposited *S. aureus* by phagocytes, demonstrating that eLtaS aggravates infection by mediating evasion of phagocytosis by C3b*-*deposited *S. aureus.*

Furthermore, we demonstrated that the mouse monoclonal antibody MAE4 inhibited the interaction between eLtaS and C3b and consequently blocked evasion of phagocytosis of C3b-deposited *S. aureus. In vivo* studies also showed that administration of MAE4 promoted engulfment of *S. aureus* by phagocytes and protected mice against challenge with drug-sensitive (8325-4, Newman) and drug-resistant (04018) strains of *S. aureus*.

Our results *in vitro* showed that MAE4 did not target LtaS located on the surface of *S. aureus*; moreover, it had no effect on LTA synthesis and growth of *S. aureus*, suggesting that MAE4 could not inhibit the activity of membrane-bound LtaS. No observed interaction between MAE4 and LtaS on *S. aureus* may be because the epitope on LtaS recognized by MAE4 was covered. Whether MAE4 could directly target LtaS and has the inhibitory effect on *S. aureus* growth and LTA synthesis *in vivo* will be addressed in the future.

Of note, *S. aureus* expresses several small secreted proteins (~10–15 kDa) that bind to C3b, such as staphylococcal complement inhibitors (SCINs) and Efb^39^. These proteins share a similar structure for interaction with C3b^39^. Compared with these proteins, eLtaS is larger (~50 kDa) and a competitive ELISA also indicated that the C3b-binding site of eLtaS was different.

In conclusion, we have shown that the secreted protein eLtaS is an important virulence factor of *S. aureus* that mediates immune evasion by interfering with phagocytosis of complement-deposited bacterial cells. Targeting of eLtaS using the neutralizing antibody MAE4 that we have generated is a potential therapeutic strategy for treatment of infectious diseases caused by *S. aureus*.

## Methods

### Ethics Statement

All animal experimental protocols of the study are in accordance with the national guidelines for the use of animals in scientific research “Regulations for the Administration of Affairs Concerning Experimental Animals” and were approved by the Animal Care and Use Committee of Beijing Institute of Basic Medical Sciences, with the approval number BMS-111248.

### Bacterial strains and growth conditions

*S. aureus* strains were grown in 5 ml of brain heart infusion (BHI) (BD) at 37 °C for 12 h with shaking at 200 rpm.

### Hemolysis assay

For preparation of bacteria-free culture supernatant, a single colony was used to inoculate 5 ml of BHI in a 50-ml conical tube. The cultures were incubated at 37 °C with shaking at 200 rpm for 6 h and 12 h, at which time the samples were placed on ice. The cultures were diluted with BHI to equalize the OD_600_ to a value at 10, pelleted by centrifugation at 4 °C, and sterile filtered through a 0.2-μm filter. The classical pathway-mediated hemolytic assay was performed as previously described with some modifications^40^. Briefly, normal human sera were first incubated with sheep erythrocytes to pre-clear serum of antibodies directed against erythrocytes. Concurrently, sheep erythrocytes were opsonized with anti-erythrocyte IgM. The opsonized sheep erythrocytes (2 × 10^7^) were then incubated with 25% precleared normal human serum, in the presence of eLtaS or the supernatants of *S. aureus* 8325-4 or Δrnc in HBS^++^ buffer (20 mM HEPES, 140 mM NaCl plus 5 mM CaCl_2_, 2.5 mM MgCl_2_ and 0.1% Tween-20, pH 7.4). After 30 min at 37 °C, the samples were centrifuged, and the absorbance of the supernatants at 405 nm was measured.

### eLtaS protein expression and purification

The *eltaS* gene was amplified by PCR from the genomic DNA of *S. aureus* 8325-4 using the primer sequences (forward-Eco*RI*) ccggaattctctgaagatgacttaacaaa and (reverse-Xho*I*) ccgctcgagttattttttagagtttgctt. The PCR fragment was subcloned into the expression vector pET-28(a) and expressed in *Escherichia coli* (BL21) as an N-terminal his-tag fusion protein. The fusion protein was purified by Ni-NTA agarose (Qiagen). Purified eLtaS protein was passed through the pierce high-capacity endotoxin removal resin to remove residual *E. coli* endotoxins (Thermo Scientific). Protein concentrations were determined by use of the bicinchoninic acid (BCA) protein assay, and proteins were stored at −70 °C until use.

### Complement Inhibition Assays

Complement inhibition assays were performed as previously described^22^. Briefly, the lectin pathway and alternative pathway were assessed by using immobilized mannan (10 μg/well, *Saccharomyces cerevisiae*, M7504, Sigma) and LPS (1 μg/well, *Salmonella enteriditis*, L6386, Sigma) respectively, as ligands. Human fibrinogen (10 μg/well, human plasma, F3879, Sigma) was used for assessing the classical pathway. Then, antibodies against fibrinogen (Merck, 1:1000) were added to generate Immune complexes. Plates were blocked with 5% skimmed milk/PBS for 1 h at 37 °C. Serum samples were diluted in GVB^++^ (VBS containing 0.5 mM MgCl_2_, 2 mM CaCl_2_, 0.05% Tween-20 and 0.1% gelatin, pH 7.5) to assess the classic and the lectin pathway, while GVB^+^ (VBS containing 10 mM EGTA, 5 mM MgCl_2_, 0.05% Tween-20 and 0.1% gelatin, pH 7.5) were used to assess the alternative pathway. To evaluate the effect of eLtaS, serum samples were pre-incubated with eLtaS for 10 min at room temperature before adding them to the plates for 1 h at 37 °C. Deposited C3b and the formation of C5b-9 were detected using anti-C3 (Cedarlane, 1:1000) and anti-C5b-9 (Abcam, 1:1000) antibodies respectively, followed by peroxidase (HRP)-conjugated goat anti-mouse IgG. The absorbance at 450 nm was measured. All incubation volumes were 100 μl and detection antibodies were diluted in PBS containing 5% skimmed milk. After each step, plates were washed three times with PBS containing 0.5% Tween-20.

### Enzyme-linked immunosorbent assay

Capture ELISA was performed as previously described with some modifications^29^. Plates were coated with eLtaS (5 μg/well). The human or mouse sera were diluted in GVB-EDTA buffer (VBS containing 0.1% gelatin and 40 mM EDTA) and then added for 1 h at 37 °C. Detection of C3 was performed using anti-C3 (Cerdalane, 1:1000), followed by HRP-conjugated goat anti-rabbit IgG.

The binding ELISA was performed as previously described with some modifications^22^. Human complement C3 and its fragments C3b and C3d (Merck Millipore) were coated onto ELISA plates (1 μg/well). eLtaS protein was diluted in PBS and then added for 1 h at 37 °C. Bound eLtaS was detected using anti-eLtaS antibody (1:1000) followed by HRP-conjugated goat anti-rabbit IgG.

In the eLtaS competitive ELISA, CR1 protein (USCN) was coated at 1 μg per well; then 1 μg C3b protein was added to each well with a serial dilution of eLtaS protein. Bound C3b proteins were detected using anti-C3 (Santa Cruz Biotechnology) followed by HRP-conjugated goat anti-rabbit IgG (1:20,000, Jackson ImmunoResearch Laboratories).

In the MAE4 competitive ELISA assay, eLtaS was coated at 1 μg per well; then 2 μg C3b was added to each well with a serial dilution of MAE4. Bound C3b proteins were detected using anti-C3 (Santa Cruz Biotechnology) followed by HRP-conjugated goat anti-rabbit IgG (1:20,000, Jackson ImmunoResearch Laboratories).

### Acute peritoneal infection murine model

Male mice (CD-1, C3^−/−^ C57BL/6 and C3^+/−^ C57BL/6; 6- to 8-week-old) were intraperitoneally injected with *S. aureus* 8325-4 or Δ*ltaS* cells. Each type of mice was randomized into treatment groups of eight mice each. In the eLtaS or MAE4 group, 100 μg protein/per mouse carried in 100 μl PBS was injected into the peritoneal cavity. Mouse survival was recorded at the different time points post challenge.

### Sublethal murine pneumonia infection model

Male CD-1 mice, 6- to 8-week-old, were challenged with *S. aureus* 8325-4 (1 × 10^7^ cfu/mouse) via the tracheal route and were randomized into two groups of eight mice each. eLtaS or MAE4 protein (100 μg per mouse in 300 μl PBS) was intraperitoneally injected. After 72 h of challenge, the animals were sacrificed and the lung tissues were fixed and stained by H&E.

### Renal abscess model and lethal challenge via intravenous injection

BALB/c mice (8-week-old, female) were intraperitoneally injected with 100 μg antibody/per mouse in 100 μl PBS. Mice were injected retro-orbitally with clinical *S. aureus strain* Newman (1 × 10^7^ cfu) under anesthesia. On the 5th day post infection, mice were killed and the right kidneys were examined by histopathology for internal abscesses. Left kidneys were removed and homogenized in 0.1% Triton X-100. Aliquots were diluted and plated on agar medium for triplicate determination of cfu. For the lethal challenge model, all experimental conditions remained the same except that 1 × 10^8^ cfu were administered and animals were monitored for survival for ten days post infection.

### SYTO 9 labeling *S. aureus*

*S. aureus* 8325-4 was labeled by SYTO9 as previously described with some modifications^41^. Briefly, *S. aureus* 8325-4 was grown on BHI for 12 h at 37 °C. The bacterial cells were collected by centrifugation (5,000 *g*, 5 min), washed with PBS, suspended in PBS to an optical density of 2 × 10^9^ cfu/ml, and incubated at room temperature for 30 min in the dark with 5 μM SYTO 9 (Molecular Probes). The cells were washed twice to remove excess dye and resuspended in PBS. A flow cytometer (BD) with a 530/30 band pass filter was used to detect SYTO 9 fluorescence.

### *S. aureus* with C3b deposition

C3b-deposited *S. aureus* 8325-4 was prepared as described with some modifications^42^. Human sera were obtained from normal donors after informed consent, according to human study protocols reviewed and approved by the institutional review boards at Beijing Institute of Basic Medical Sciences. *S. aureus* cells (1 × 10^7^ cfu) were washed twice in PBS. A mixture of *S. aureus* cells and human serum was incubated in 100 μl (final volume) of PBS, with or without eLtaS protein, for 10 min at 37 °C. Controls for C3b deposition experiments included *S. aureus* cells incubated with buffer alone and eLtaS alone.

Deposition of C3b was confirmed by direct immunofluorescence analysis using flow cytometry. For flow cytometric analysis, *S. aureus* cells (1 × 10^7^ cfu) with C3b deposits were resuspended in PBS, incubated with FITC-conjugated mouse anti-human C3b antibody (1 μg/ml, Cedarlane) for 30 min at room temperature, washed twice in PBS and resuspended in the same buffer at 1 × 10^6^ cells/ml.

### Assessment of phagocytosis *in vitro*

The C3b-deposited *S. aureus* 8325-4 (5 × 10^6^ cfu) was incubated with different concentrations of eLtaS for 30 min at room temperature. For colony-forming unites assay, human neutrophils were purified on a Ficoll (Amersham)/Histopaque (density 1.119; Sigma) gradient using heparinized whole blood from a single donor^30^.

Then the C3b-deposited *S. aureus* 8325-4 with or without eLtaS were incubated with human neutrophils for 10 min at 37 °C. The reaction was stopped by adding gentamicin (0.1 mg/ml) for 10 min at 37 °C. The bacteria were resuspended in 0.02% Triton X-100. Cfu were calculated by serial dilution plated on Todd-Hewitt agar (THA; 1.5% agar in Todd-Hewitt broth) plates.

For flow cytometry assays, leukocytes were isolated from whole blood by red blood cell lysis. The C3b-deposited *S. aureus* 8325-4 (labeled with SYTO 9) with eLtaS were incubated with leukocytes for 10 min. The cells were washed twice by PBS and the percentage of granulocyte cells that engulfed the SYTO 9-labeled *S. aureus* was detected by flow cytometry.

### Assessment of phagocytosis *in vivo*

SYTO9-labeled *S. aureus* 8325-4 (5 × 10^8^ cfu/mouse) were intraperitoneally injected into individual mice together with eLtaS (100 μg/mouse) in 500 μl PBS. Peritoneal cavity cells were isolated 30 min after injection. The reaction between peritoneal cavity cells and *S. aureus* was stopped by adding gentamicin (0.1 mg/ml) for 10 min at 37 °C. After washing two times with PBS, cell suspensions were then preincubated with anti–CD16/CD32 mAb to block FcγRII/III receptors and stained in 4 °C for 15 min. Cells were then stained with APC-conjugated anti-CD11b (Mac-1) (M1/70), PE-conjugated anti-F4/80 (BM8) and PercP-Cy5.5-conjugated Gr-1(Ly-6 G) (1A8) (Biolegend) in 4 °C for 30 min. For dead cell exclusion, stained cells were resuspended in 5 μg/mL 7-AAD to exclude dead cells. Data were collected from 1 to 5 × 10^5^ cells and analyzed with FlowJo software. To distinguish autofluorescent cells from cells expressing low levels of individual surface markers, we established upper thresholds for autofluorescence by running an unstained sample. Single-stain controls are then analysed to determine the correct voltage and gain settings.

### Production and characterization of monoclonal antibodies

Monoclonal antibodies of eLtaS were produced by fusing antibody-secreting spleen cells from eLtaS-immunized mice with immortal myeloma cells to create monoclonal hybridoma cell lines that express the eLtaS specific antibody in cell culture supernatant. Antibody samples from cell culture supernatants were screened to identify eLtaS-binding specificity. The titer and isotype of positive antibody samples were determined. To produce large quantities of antibody MAE4, the MAE4 hybridoma cells were injected into the abdomen of mice where the cells multiplied and produced antibody-filled fluid (ascites). The antibodies were purified using protein G agarose (GE Healthcare) according to the product instructions.

### Statistical analysis

All experiments were repeated at least three times and the data were presented as the mean ± SD unless noted other wise. Significant differences between groups were evaluated using a two-tailed Student’s t test. Survival curves were determined using the Kaplan-Meier method and compared using the log-rank test. A P-value of less than 0.05 was considered statistically significant. GraphPad Prism software was used for statistical analyses.

## Additional Information

**Accession codes:** National Center for Biotechnology Information (http://www.ncbi.nlm.nih.gov): ltaS, GI:87201381.

**How to cite this article**: Liu, Y. *et al.* MAE4, an eLtaS monoclonal antibody, blocks *Staphylococcus aureus* virulence. *Sci. Rep.* **5**, 17215; doi: 10.1038/srep17215 (2015).

## Supplementary Material

Supplementary Information
