# Supplementary material for: MAE4, an eLtaS monoclonal antibody, blocks Staphylococcus aureus virulence
Source: Sci Rep. 2015 Nov 24;5:17215. doi: 10.1038/srep17215 (PMC4657049; doi:10.1038/srep17215)
Supplement: Supplementary Information [file srep17215-s1.pdf]

**Title**

# MAE4, an eLtaS monoclonal antibody, blocks *Staphylococcus aureus* virulence

**Authors:**

Yu Liu[†1,2], Jiannan Feng[†1,2], Qiang Lu[1], Xin Zhang[3], Yaping Gao[1,2], Jun Yan[1,2], Chunhua Mu[1], Yan Hei[4], Ming Lv[1,2], Gencheng Han[1,2], Guojiang Chen[1,2], Peng Jin[1], Weiguo Hu[3], Beifen Shen[1,2], Guang Yang*[1,2]

**Affiliations:**

[1]Beijing Institute of Basic Medical Sciences, Beijing, China

[2]State key Laboratory of Toxicology and Medical Countermeasures

[3]Institutes of Biomedical Sciences, Fudan University, Shanghai, China

[4]People's Armed Police Corps General Hospital, Beijing, China

[†]These authors contributed equally to this work.

*Corresponding author: Guang Yang, Ph.D., E-mail: yangg62033@163.com, Tel/Fax: 86-10-68163140(O)

# Supplementary files

## Figure S1

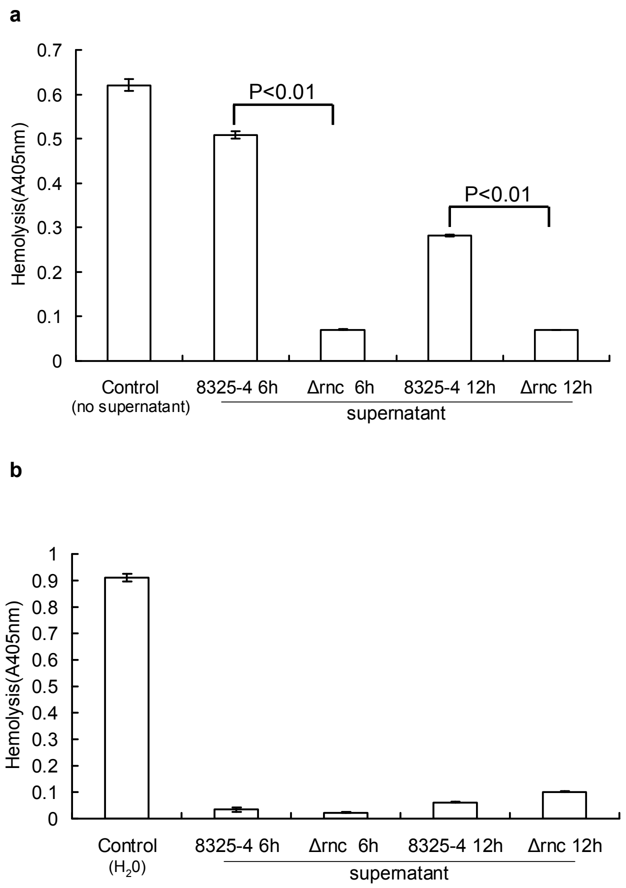


**Figure S1. The supernatant of the Δrnc strain significantly inhibits complement activation.** Opsonized sheep erythrocytes ($2 \times 10^7$) were incubated with (**a**) or without (**b**) 25% pre-cleared normal human serum in the presence of supernatants of *S. aureus* 8325-4 or Δrnc cultured for 6 and 12 h. After 30 min at 37°C, the samples were centrifuged, and the absorbance of the supernatants was measured at 405 nm.

## Figure S2

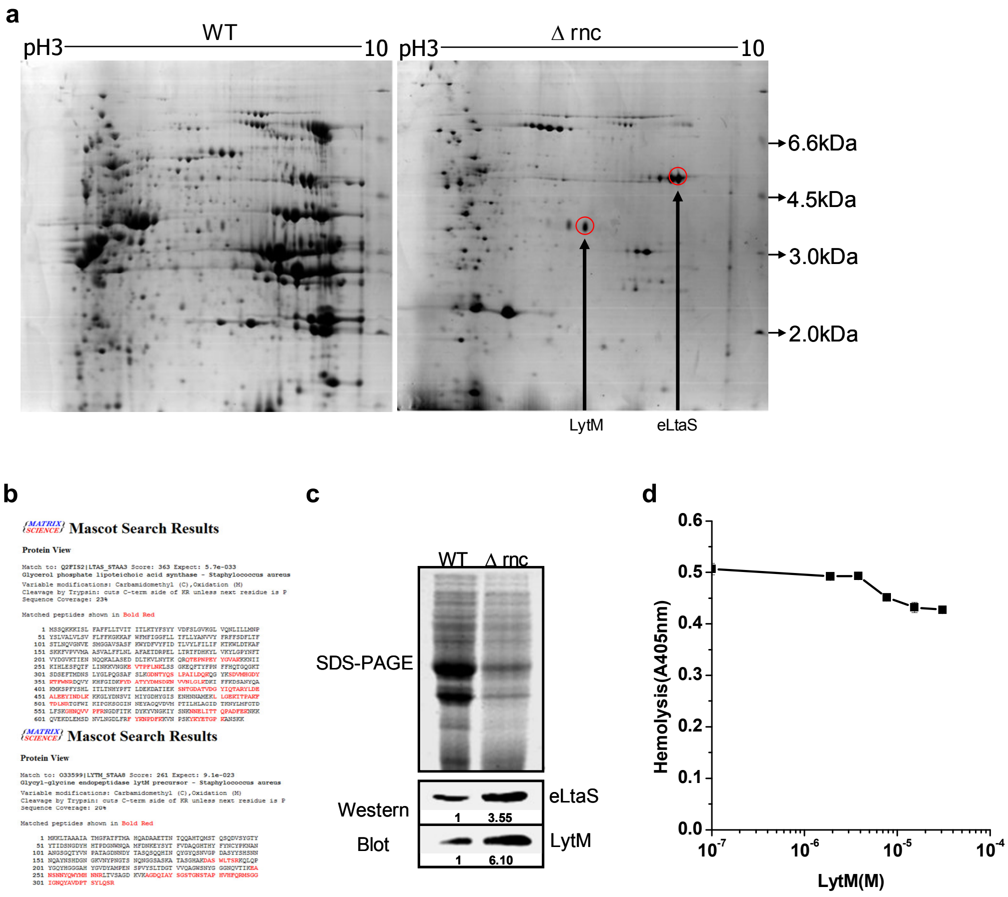


**Figure S2. The level of eLtaS is elevated in the Δrnc strain. (a)** Two-dimensional gel electrophoresis of the supernatant of the WT (8325-4) and Δrnc strains. Culture supernatants from equal numbers (1 × $10^9$ CFU) of *S. aureus* 8325-4 and Δrnc were precipitated by adjusting the filtered supernatants to 10% tricarboxylic acid (TCA) and incubating at 4°C for 4 h. Precipitated proteins were separated on preparative two-dimensional gels. Two proteins were expressed at higher levels in the Δrnc strain (indicated by arrows). **(b)** The two proteins expressed at higher levels in the Δrnc strain were identified by matrix-assisted laser desorption ionization–time of flight mass spectrometry. Matched peptides are highlighted in bold red. **(c)** The levels of eLtaS and LytM protein present in the supernatant were determined by western blotting. Culture supernatant proteins from equal numbers (1 × $10^9$ CFU) of *S. aureus* 8325-4 and Δrnc were precipitated and separated on SDS-PAGE. The levels of eLtaS and LytM were analyzed by western blotting using antibodies specific to eLtaS and LytM, respectively. **(d)** Detection of the effect of LytM on red blood cell lysis mediated by the complement system. Opsonized sheep erythrocytes (2 × $10^7$) were incubated with 25% pre-cleared normal human serum in the presence of LytM at the concentrations indicated for 30 min at 37°C. The samples were centrifuged, and the absorbance of the supernatants was measured at 405 nm.

Figure S3

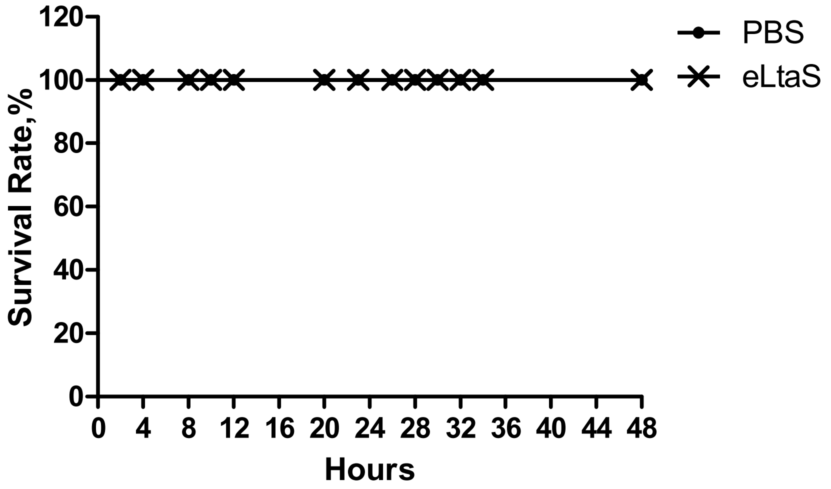


**Figure S3. Detection of eLtaS toxicity.** eLtaS (100 μg/mouse) was injected into the abdominal cavity of CD-1 mice. The survival rate at different time points was recorded.

Figure S4

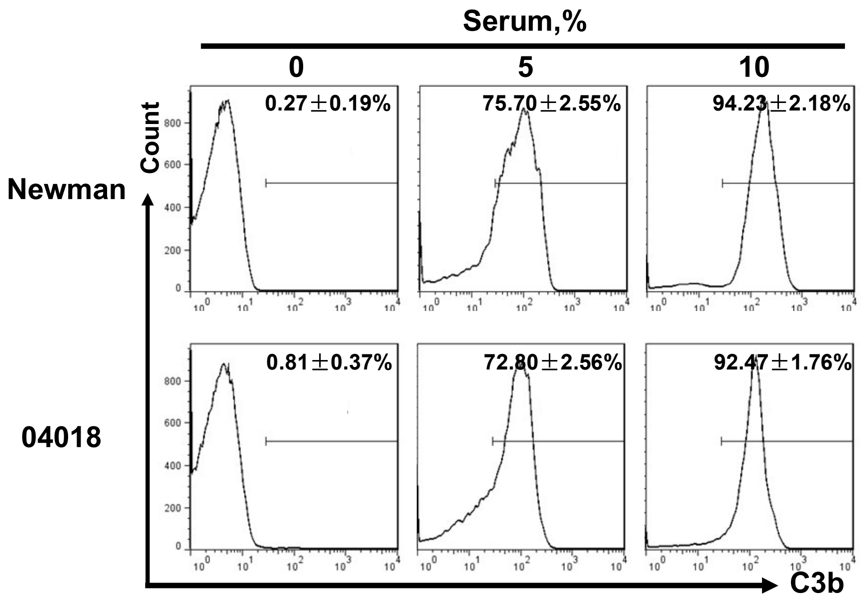


**Figure S4. Detection of the deposition of C3b on the bacterial surface.** *S. aureus* Newman and 04018 were incubated with human serum for 15 min. After washing with PBS, the amount of deposited C3b was determined by FCM using an anti-C3b antibody.

**Figure S5**

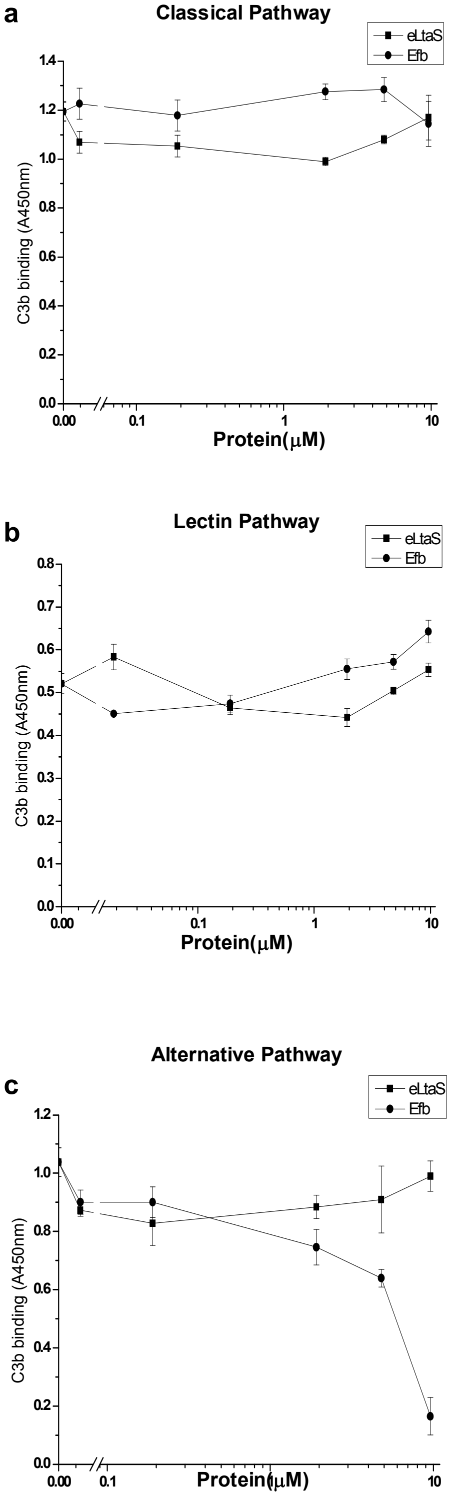


**Figure S5. Determination of the effect of eLtaS on C3b deposition in the three complement activation pathways.** Serum samples were pre-incubated with eLtaS at the concentrations indicated and added to 96-well plates coated with fibrinogen immune complex (classic pathway), immobilized mannan (lectin pathway) or LPS (alternative pathway). The deposition of C3b was determined using an anti-C3b antibody.

**Figure S6**

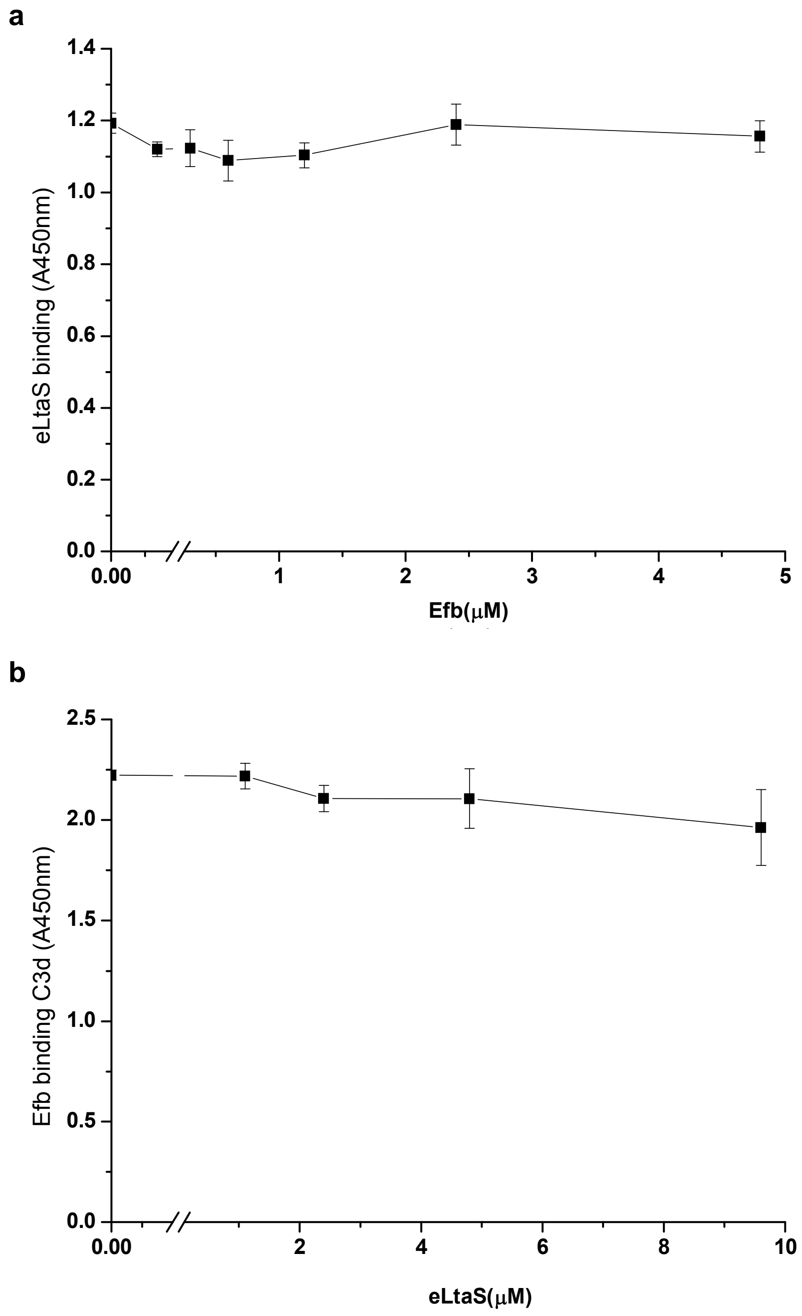


**Figure S6. The interaction between eLtaS and C3d is not inhibited by Efb. (A)** C3d (1 μg/well) was incubated with eLtaS prior to the addition of Efb (4.8, 2.4, 1.2, 0.6, 0.3, 0.15 μM). Binding was measured using anti-eLtaS antibodies. **(B)** C3d (1 μg/well) was incubated with Efb prior to the addition of eLtaS (9.6, 4.8, 2.4, 1.2 μM). Binding was measured using anti-Efb antibodies. Results are represented as the mean of three independent experiments.

**Figure S7**

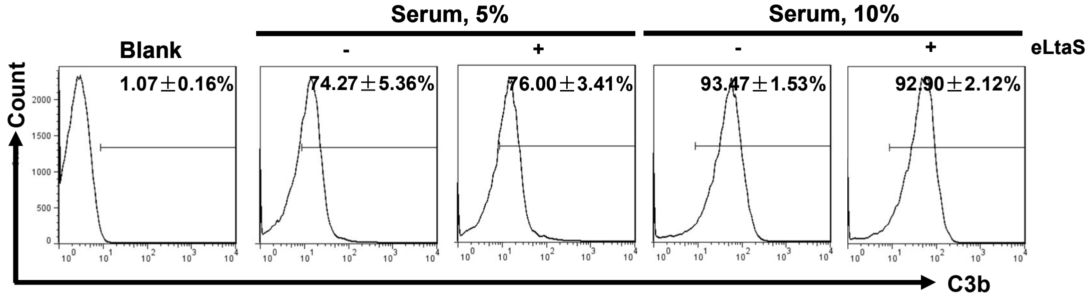


**Figure S7. Determination of the effect of eLtaS on C3b deposition on the surface of *S. aureus.*** Different concentrations of human serum were incubated with S. aureus in the presence of eLtaS (10 μg/ml) for 15 min. The deposition of C3b was determined by FCM using an anti-C3b-FITC antibody.

Figure S8

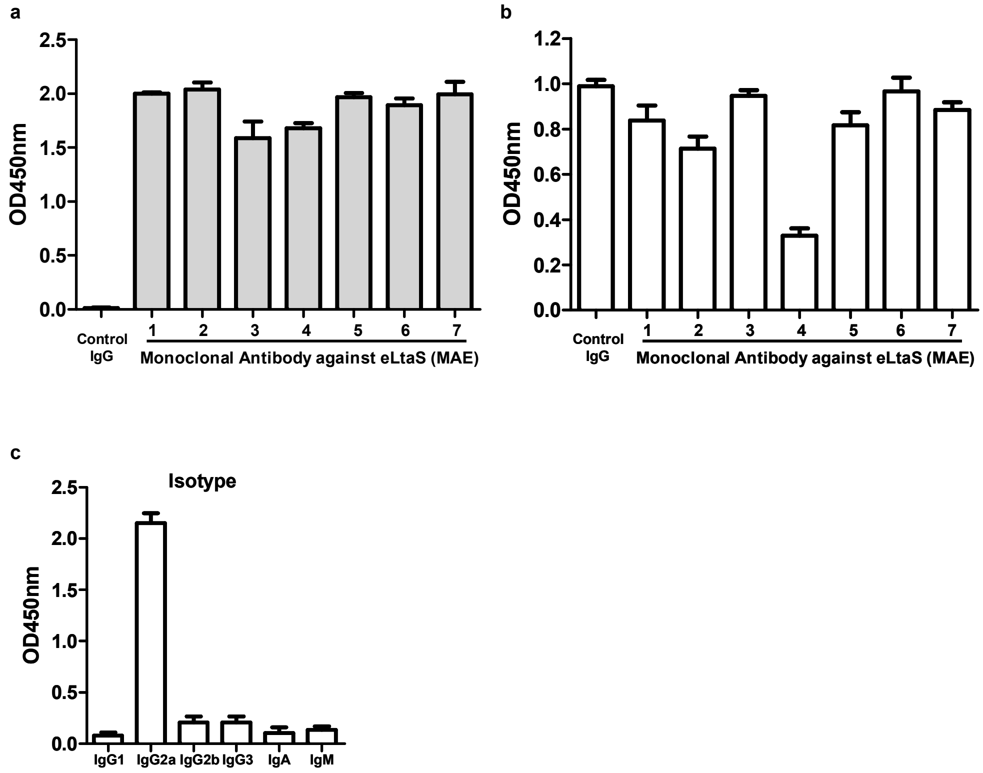


**Figure S8. Production and characterization of monoclonal antibodies against eLtaS. (a)** Detection of the interaction between eLtaS and seven monoclonal antibodies by ELISA. **(b)** Screening of monoclonal antibodies for the ability to inhibit the binding of eLtaS to C3b. **(c)** The isotype of MAE4 to eLtaS was determined by ELISA. eLtaS protein (1 μg/well) was coated onto 96-well plates in the presence of 100 ng of MAE4. Bound IgG was detected using (HRP)-conjugated goat anti-mouse IgG1, IgG2a, IgG2b, IgG3, IgA, or IgM antibodies.

**Figure S9**

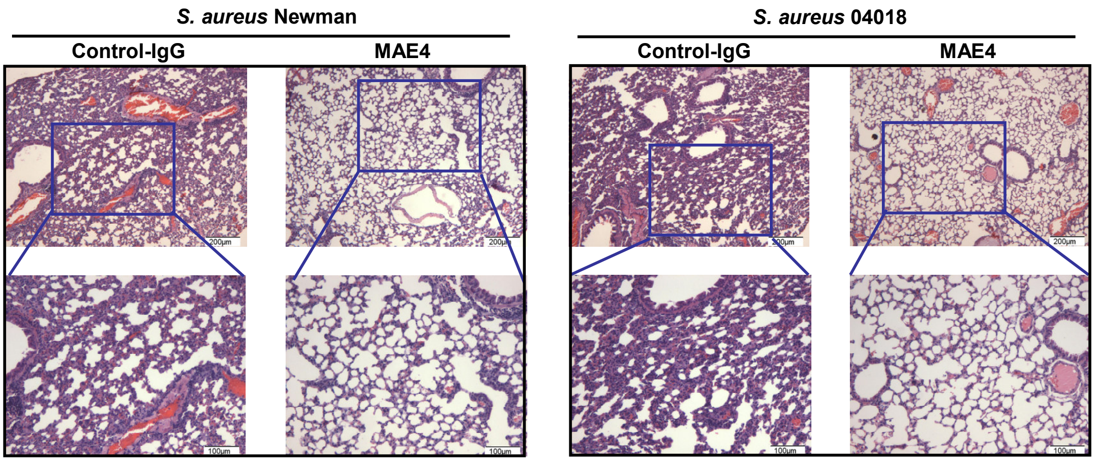


**Figure S9. MAE4 protects mice from *S. aureus* infection in a pneumonia infection model using the clinical *S. aureus* strains Newman and 04018 for challenge.** CD-1 mice were injected intratracheally with *S. aureus* cells ($1 \times 10^7$ cfu/mouse). MAE4 IgG (100 µg/mouse) was injected intramuscularly into the left hind leg at 30 min, 24 h, and 48 h post infection. Lung sections were obtained 72 h post challenge and stained with hematoxylin-eosin.

**Figure S10**

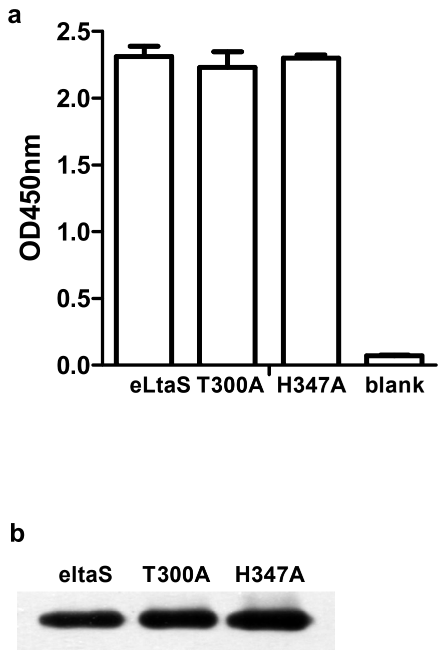


**Figure S10. MAE4 recognized eLtaS and two eLtaS-derived mutant proteins.** The interaction between MAE4 and proteins (eLtaS, T300A, H347A) was detected by ELISA (**a**) and western blotting (**b**).

**Table S1**

| Target | Analyte | $K_{on}(M^{-1}s^{-1})$ | $K_{dis}(s^{-1})$ | $K_D$ (M) |
|---|---|---|---|---|
| eLtaS | C3 | $2.17X10^6$ | $1.49X10^{-3}$ | $6.87X10^{-10}$ |
| eLtaS | C3b | $3.63X10^6$ | $1.98X10^{-3}$ | $5.46X10^{-10}$ |

**Table S1. Summary of the kenitics ($K_{dis}$,$K_{on}$) and affinity($K_D$) results for binding of eLtaS and human C3 or C3b.** The biotinylated eLtaS protein was immobilized in five channel of Sensor Chip SA. C3 and C3b were prepared at concentrations of 300, 100, 30, 10 μg/ml and injected into the analyze channel. Global kinetic rate constants ($K_{on}$ and $K_{dis}$) were derived from the interaction of the different C3 or C3b concentrations with one concentration of immobilized eLtaS.
